# The Lipotubuloids of *Ornithogalum umbellatum* L. Contain Hyperstable Microtubules

**DOI:** 10.3390/plants14233677

**Published:** 2025-12-03

**Authors:** Krithika Yogeeswaran, Manfred Ingerfeld, Nicholas R. McInnes, Brian E. S. Gunning, David A. Collings

**Affiliations:** 1 School of Biological Sciences, University of Canterbury, Private Bag 4800, Christchurch 8140, New Zealandmanfredi@xtra.co.nz (M.I.); 2 Research School of Biology, Australian National University, Canberra, ACT 2601, Australiabrian.gunning@anu.edu.au (B.E.S.G.); 3 School of Environmental and Life Sciences, University of Newcastle, Callaghan, NSW 2308, Australia

**Keywords:** hyperstable microtubules, lipid droplets, lipotubuloids, microtubules, microtubule-associated proteins, *Ornithogalum*, oryzalin, transient expression

## Abstract

The epidermal cells of bracts, petals and sepals of *Ornithogalum umbellatum* L. (Star-of-Bethlehem, Asparagaceae) contain lipotubuloids, complex aggregates of lipid droplets (LDs) enmeshed by bundles of microtubules (MTs). We investigated lipotubuloid organization and stability through the transient expression of GFP fusion proteins targeted to different subcellular structures and with immunofluorescence and transmission electron microscopy (TEM). Live cell imaging confirmed that lipotubuloids contain LDs, organelles including endomembranes, mitochondria and peroxisomes, a tonoplast-defined vacuole, and that they move through actin microfilament-based streaming. Intriguingly, the different microscopy modes used showed different patterns of MT organization in the lipotubuloid. While MT sheets and bundles were visible by TEM, few MTs were seen with fusion proteins and immunofluorescence. Oryzalin-based MT depolymerization experiments suggest a possible resolution for this paradox: TEM showed that lipotubuloid MTs resisted depolymerization, even after 20 h in oryzalin, while MT polymerization was visible in lipotubuloids with fusion proteins during oryzalin wash-out. These results suggest that the *Ornithogalum* lipotubuloids contain hyperstable MTs, possibly formed with microtubule-associated proteins (MAPs) that normally occlude fusion protein and antibody binding sites.

## 1. Introduction

Lipid droplets (LDs) in plant cells, otherwise known as lipid bodies, oil bodies or oil droplets, contain neutral lipids, predominantly triacylglycerides and sterol esters, and are surrounded by a phospholipid monolayer. LDs are prominent in seeds and pollen grains where they provide an energy source for seedling germination and pollen tube growth [1,2] but also occur in many other tissues [3]. As it would be energetically favorable for small LDs to coalesce into larger LDs, factors must be present that prevent this. In seeds and pollen, oleosin proteins stabilize LDs by coating the LD surface, but these proteins are typically absent from vegetative tissues where caleosin and steroleosin proteins coat LD surfaces [1,2,4].

The LDs of seeds have been extensively studied because of their importance to agriculture. However, LDs in other tissues are not only less common but also less well studied. In leaf epidermal cells, LDs can accumulate in response to abiotic stresses such as heat and drought suggesting that they may play some protective function [1,5]. Additionally, it has been speculated that LDs in epidermal cells may contribute to the formation of the cuticle because this process requires the extensive synthesis of fatty acids [6,7].

One unusual example of LD aggregates occurs in the epidermal cells of the ovaries, floral bracts, petals and sepals of the monocot *Ornithogalum umbellatum* [8]. In a series of more than twenty papers published over four decades from 1966 [9], the Polish botanist Maria Kwiatkowska investigated the LD aggregates in the *Ornithogalum* ovary epidermis. Transmission electron microscopy (TEM) demonstrated that these aggregates comprised small LDs with a diameter of ~400 nm that are embedded in a network of microtubules (MTs), and that the aggregates also included other organelles including endoplasmic reticulum (ER), Golgi apparatus and mitochondria [6]. Based on the combination of lipids and MTs, the name lipotubuloid was proposed [10]. The lipotubuloids, while structurally stable, show comparatively rapid movement within the cell showing, at times, both streaming and rotations [11,12] with this motion dependent on actin microfilaments [13]. The number of lipotubuloids varies between species, with multiple lipotubuloids present in *O. caudatum* [12], whereas there is typically only a single lipotubuloid in each cell of *O. umbellatum* [10,14]. The function of lipotubuloid remains unknown. Radiotracer studies have suggested that the lipotubuloids are sites of lipid biosynthesis [15] and that they may be involved in biosynthesis of the cuticle [16,17], with this supported by enzymatic evidence including the presence of glycerol-3-phosphate acyltransferase6 (GPAT6) and diacylglycerol acyltransferase 2 (DGAT2) [18]. The function of the lipotubuloid MTs is also unknown, although a role in lipid synthesis has been proposed [19]. It is also possible that the presence of MTs bound to the surface of the LDs physically blocks their coalescence into larger droplets, something that might be energetically favorable, ensuring an increase in their overall surface area.

Previous investigations of the *Ornithogalum* lipotubuloid have used fixed tissue and TEM, but in this study, we have reinvestigated the structure of the lipotubuloid using a combination of TEM, immunofluorescence, video microscope and confocal imaging of GFP fusion proteins in living cells. We studied lipotubuloids in the abaxial epidermis of flower petals, sepals and floral bracts as these were more readily accessible for live cell imaging and the transient expression of GFP constructs. Our observations confirm that the lipotubuloid is a complex aggregation of LDs, MTs and organelles including endoplasmic reticulum, Golgi vesicles, mitochondria and peroxisomes, and that the movement of lipotubuloids depends on actin microfilaments. MT disruption with oryzalin does not modify lipotubuloid structure and causes only limited MT depolymerization within the lipotubuloid. This suggests that the MTs in the lipotubuloid might be more stable than those in the cell cortex.

## 2. Results

### 2.1. Microtubules Are a Major Component of Lipotubuloids

**Figure 1:**
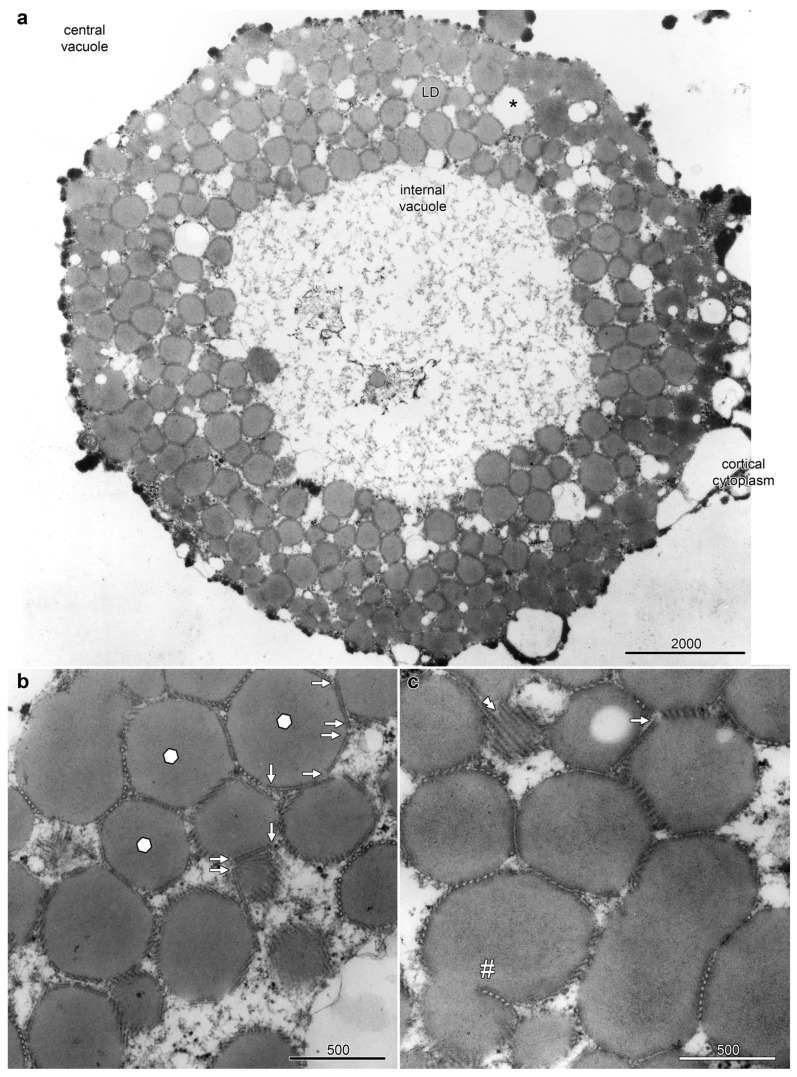
Lipotubuloids were composed of small lipid droplets (LDs) enmeshed by MTs. (**a**) A single lipotubuloid, situated between the cortical cytoplasm and central vacuole, was hollow with an internal vacuole at its center. Other membrane-bound organelles were occasionally present (asterisk). (**b**) MT formed sheets that enmeshed the LDs, often compressing the LD into polyhedra with flattened surfaces (marked with hexagons). MTs often ended abruptly (arrows). (**c**) A sheet of MTs (double arrowheads) and a sheet of MTs present in a deep cleft formed in a single LD (hashmark). Bars: (**a**) 2000 nm; (**b**,**c**) 500 nm. Images modified from [12].

The epidermal cells on the abaxial side of the petals, sepals and floral bracts of *Ornithogalum umbellatum* contained distinctive and complex aggregations of LDs, referred to as lipotubuloids. Most epidermal cells had a single, spherical lipotubuloid, about 10–25 μm in diameter, composed of smaller particles. TEM confirmed the ultrastructure of the lipotubuloids showing that these were primarily composed of LDs (Figure 1); although, other membrane-bound organelles were sometimes present (asterisk). Sheets of coaligned, closely packed MTs were present on the faces of the LDs (Figure 1a, LD). Individual MTs varied in length, some with abrupt termini (Figure 1b) and some extending in the plane of section from one LD to another, adjacent one. Where the MT sheets abut LDs, the normally spherical surface of the LDs were flattened so that in section they appeared as polyhedra, with differently oriented MT sheets on each facet, sometimes being seen running in the plane of section or in cross section but more often oblique. Other LDs were highly elongated, while MT sheets were also present in deep clefts within individual LDs (Figure 1c, hashmark). These observations all suggest that the LDs are subject to compressive forces and that the MTs may prevent them from coalescing.

**Figure 2:**
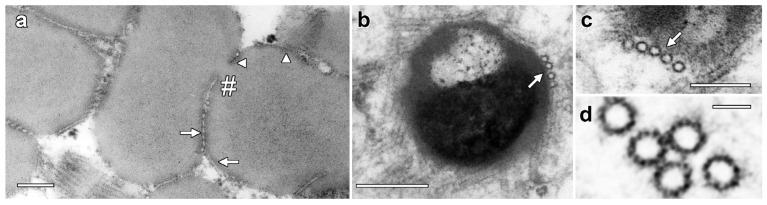
Higher-resolution images of MT associated with lipotubuloids. (**a**) Enhanced density was seen on the surface of LDs where MTs were appressed (arrow) but also where they were absent (arrowheads). MTs were also present in the deep cleft in a partially separated pair of lipotubuloids (hashmark). (**b**,**c**) MTs were often associated with the LD surface (arrows). (**d**) Internal structure in MTs suggested 13-fold symmetry. Bars: (**a**,**b**) 200 nm; (**c**) 100 nm; (**d**) 25 nm.

Higher-magnification images showed enhanced staining in the periphery of LDs where MTs are closely appressed (Figure 2a, arrow) but also in areas where MT were absent (arrowheads). Higher-magnification images also showed an average MT diameter of 24.5 ± 0.9 nm and 13-fold symmetry, but cross-bridges were not visible between the MTs (Figure 2b,c). Serial TEM sections demonstrated consistency of LD structure and MT organization between sections (Figure S1).

### 2.2. Live Cell Imaging of Ornithogalum Lipotubuloids

**Figure 3:**
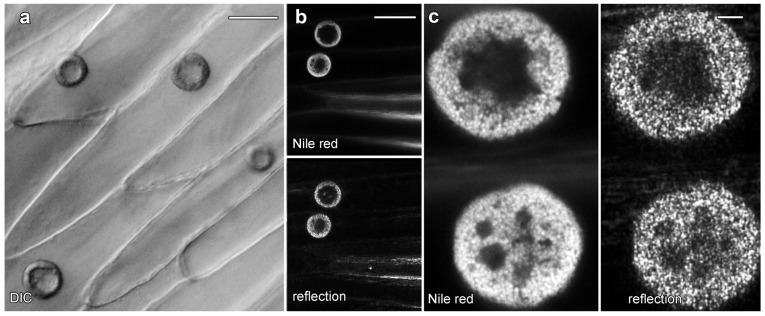
Lipotubuloids in petals of *O. umbellatum*. (**a**) Transmitted light (DIC) showed a single, prominent lipotubuloid per cell. (**b**) Confocal images revealed lipotubuloid structure with both Nile Red fluorescence and reflected light showing LDs. Adjacent cells each contained a single lipotubuloid. (**c**) Higher magnification confirmed that the lipotubuloid shell is composed of many individual LDs, also visible in the corresponding reflected light image. Bars: (**a**), 20 μm; (**b**), 50 μm; (**c**) 5 μm.

**Figure 4:**
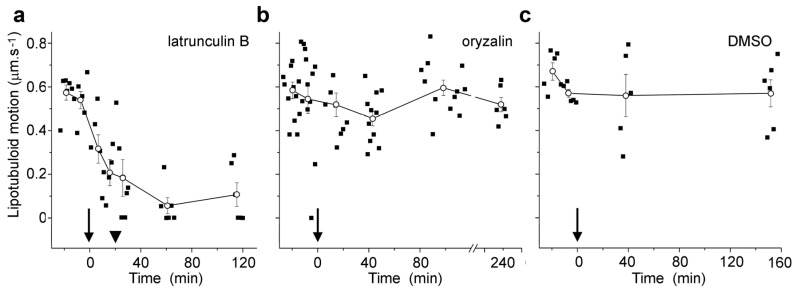
Microfilament disruption inhibited the motion of Nile Red-labeled lipotubuloids. (**a**) 1 μM latrunculin B, (**b**) 20 μM oryzalin, and (**c**) 0.5% DMSO control. Drugs were added at t = 0 min (arrows). While oryzalin and DMSO treatments were continuous, latrunculin was washed out from samples after 20 min (arrowheads). Individual data points are indicated, along with means (±standard errors, n ≥ 5 cells) for pooled data at each measurement point.

Live cell imaging was used to investigate the lipotubuloids in the abaxial epidermis of *O. umbellatum* bracts, petals and sepals. Differential interference contrast (DIC) video microscopy [12] demonstrated that the lipotubuloids show rapid cytoplasmic streaming as well as undergoing rotations (Supplementary Video S1). Rates of cytoplasmic streaming varied, typically between 0.6 and 1.0 μm·s^−1^ but sometimes as fast as 2.0 μm·s^−1^. The spherical lipotubuloids generally contained a hollow center from which particles were excluded (Figure 3a). Confocal imaging of the lipid stain Nile Red and reflected light showed that the lipotubuloids comprised small LDs (Figure 3b,c). Latrunculin B (1 μM, 15 min) rapidly and irreversibly inhibited lipotubuloid movement with little recovery of streaming even several hours after latrunculin removal (Figure 4a). Oryzalin (20 μM) resulted in no changes in lipotubuloid movement over a 4 h treatment (Figure 4b) (Supplementary Video S2).

### 2.3. GFP Labeling Reveals the Complex Array of Other Organelles in Lipotubuloids

**Figure 5:**
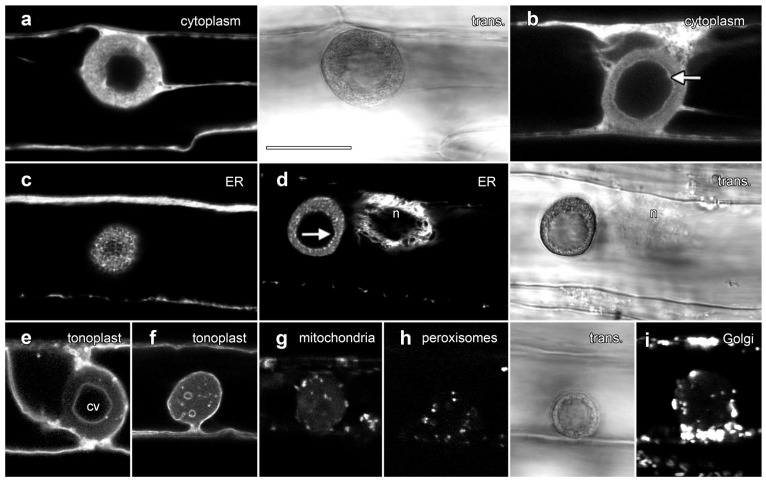
Transient expression of GFP fusion proteins showed that lipotubuloids contain numerous organelles. Images are single confocal optical sections collected concurrently with non-confocal transmitted light images (trans., shown to the right of matching fluorescence images). (**a**,**b**) Free YFP was dispersed throughout the cytoplasm but fluoresced less intensely in the lipotubuloid (**a**), while the inner edge of the outer shell of the lipotubuloid was sharply defined (arrow) (**b**). (**c**) ER-targeted GFP showed that lipotubuloids were rich in fine, reticulate ER. (**d**) ER-targeted GFP revealed that the inner edge of the lipotubuloid shell was poorly defined and uneven (arrow). n = nucleus. (**e**,**f**) Tonoplast-targeted GFP showed that lipotubuloids generally contained a central vacuole (cv) (**e**); although, some lipotubuloids contained multiple, small and scattered vacuoles (**f**). (**g**–**i**) Transient expression revealed mitochondria (**g**), peroxisomes (**h**) and Golgi apparatus (**i**) inside lipotubuloids. Bar: 10 μm for all images.

We used confocal microscopy and the transient expression of organelle-targeted GFP fusion proteins to investigate lipotubuloid organization (Figure 5). A cytosolic YFP construct labeled cortical cytoplasm and transvacuolar strands and also labeled cytoplasm within the lipotubuloids in strands that surrounded dark areas consistent with small LDs (Figure 5a). Cytoplasmic labeling showed a sharp and defined margin at the inner edge of the LD layer (Figure 5b, arrow; Supplementary Video S3). Endoplasmic reticulum-targeted GFP labeled the lipotubuloid where it revealed a delicate but stable network of ER that wrapped around individual LDs (Figure 5c; Supplementary Video S4). The inner edge of the spherical shell of the lipotubuloid was unevenly labeled (Figure 5d, arrow) in contrast to the even labeling with cytoplasmic YFP. ShMTP1-GFP labels the tonoplast and delineated the epidermal cell vacuole, confirming that the central void of the lipotubuloid is an internal vacuole surrounded by the tonoplast and that the tonoplast membrane also separated the lipotubuloid from the central vacuole (Figure 5e). Lipotubuloids without a large internal vacuole generally contained several smaller vacuoles (Figure 5f).

Fusion proteins demonstrated that three other organelles were also embedded within lipotubuloids in smaller numbers: AOX-GFP localized mitochondria (Figure 5g), GFP-SKL demonstrated peroxisomes (Figure 5h) and STtmd-GFP labeling showed numerous Golgi stacks (Figure 5i). Time-lapse imaging demonstrated not only the streaming and rotational motion of the lipotubuloids, but also that the relative positions of the internalized organelles including the mitochondria (Supplementary Video S5) and peroxisomes (Supplementary Video S6) remained stable. Imaging with plastid-targeted fluorescent proteins never indicated inclusion of this organelle into the lipotubuloids despite plastid-targeted expression being observed elsewhere within the epidermal cells.

### 2.4. GFP Constructs and Immunolabelling Struggle to Label Microtubules in Lipotubuloids

**Figure 6:**
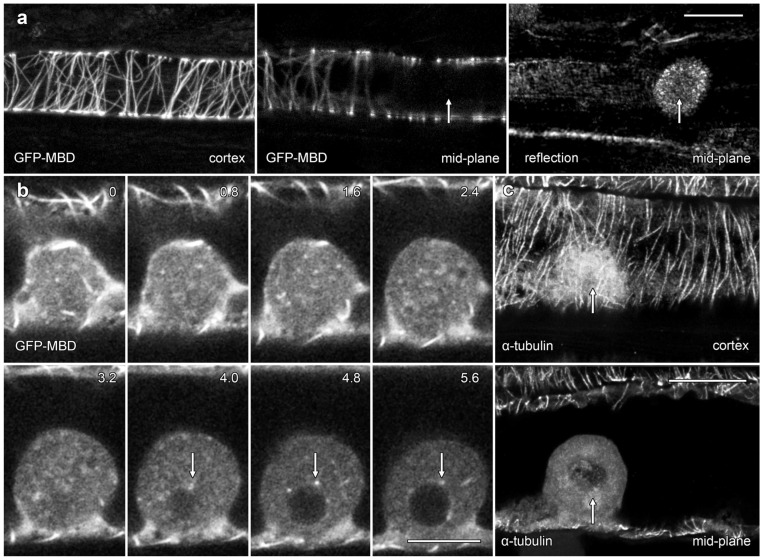
Confocal microscopy showed only rare lipotubuloid MTs even though cortical MT labeling was present. (**a**) A lipotubuloid (arrow), visible with reflected light, lacked labeled MTs although GFP-MBD showed strong labeling of cortical Ms. Two optical sections are shown, one through the cortex and the other in the cells’ midplane, along with a reflected light image. (**b**) Optical sections showing transiently expressed GFP-MBD at 0.8 μm intervals. MTs were rare, although some could be tracked between adjacent optical sections (arrows). (**c**) Immunolabeling with antibodies against α-tubulin failed to reveal MTs in the lipotubuloid (arrow) even though cortical MTs were present. Bars: (**a**,**c**) 20 μm; (**b**) 10 μm.

We used the transient expression of a GFP-tagged fusion protein and immunofluorescence microscopy to investigate the organization of MTs in lipotubuloids (Figure 6). A MT-labeling construct, GFP-MBD [20], labeled cortical MTs but only rarely labelled MTs in lipotubuloids. In many cells, lipotubuloid MT labeling was absent (Figure 6a), but in rare cases, some MTs could be tracked between optical sections (Figure 6b; arrows) (Supplementary Video S7). However, the GFP-MBD labeling never reached the density or patterning suggested by TEM. Immunofluorescence microscopy using tubulin antibodies also showed extensive cortical MT arrays in epidermal cells, but again, MT labelling was absent from lipotubuloids (Figure 6c).

### 2.5. Actin Microfilaments Are Not a Major Component of Lipotubuloids

**Figure 7:**
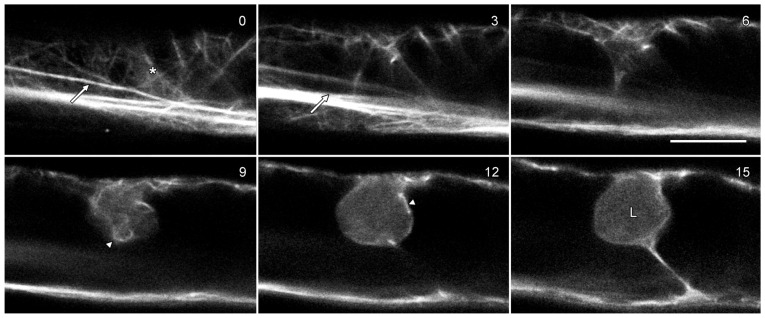
Lipotubuloids do not contain actin microfilaments. Cells were transformed with GFP-hTalin which labeled the actin cytoskeleton, primarily in long bundles (arrows) but also in a more delicate, random cortical network (asterisk) and in a basket of microfilaments surrounding the lipotubuloid (arrowheads). The lipotubuloid (L) lacked visible microfilaments. Numbers indicate the relative depth of the optical sections in micrometers. Bar: 10 μm.

Actin microfilaments have been reported in *O. umbellatum* lipotubuloids by TEM [13,19]. GFP-hTalin [21] labeled predominantly longitudinal microfilament bundles in the cytoplasm (Figure 7; arrows) with some finer and randomly oriented cortical bundles (asterisks). Few microfilaments were observed in optical sections through lipotubuloids, although lipotubuloids were surrounded by a basket of microfilaments (Figure 7; arrowheads).

### 2.6. Are Lipotubuloid Microtubules Hyperstable?

**Figure 8:**
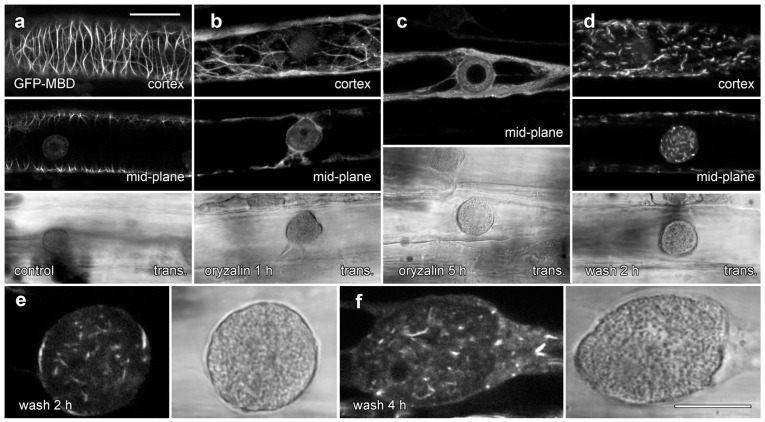
GFP-MBD labeling of lipotubuloid MTs during oryzalin wash-out. Images show GFP-MBD with corresponding transmitted light images. (**a**). Cortical MT depolymerization with oryzalin (20 µM) neared completion by 1 h (**b**) and was complete within 5 h (**c**). MT recovery 2 h after a 4 h treatment began in the cortex with the formation of short MTs (**d**). (**e**,**f**) At higher magnification, individual lipotubuloids contained short MTs during wash-out at 2 h (**e**) and 4 h (**f**). Bars: (**a**–**d**) 20 µm; (**e**,**f**) 10 µm.

**Figure 9:**
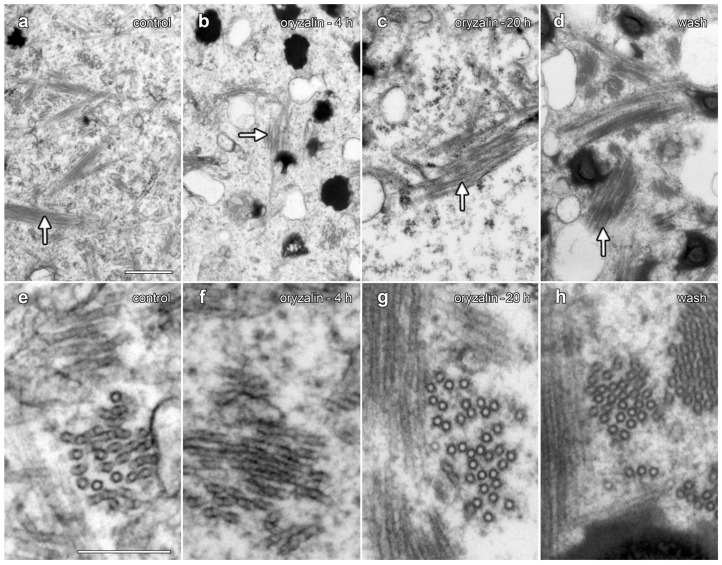
TEM demonstrated that oryzalin treatments did not depolymerize lipotubuloid MTs. (**a**–**d**) Intermediate-magnification images showed MT bundles (arrows). (**e**–**g**) Higher-magnification images. (**a**,**e**) Controls. (**b**,**f**) Oryzalin (20 µM, 4 h). (**c**,**g**) Oryzalin (20 µM, 20 h), showing little evidence of MT disruption in lipotubuloids. (**d**,**h**) Wash-out, 4 h after the removal of oryzalin (20 µM). Bar: (**a**–**d**) 500 nm; (**e**–**h**) 200 nm.

We tested the effects of 20 µM oryzalin treatments on lipotubuloid structure and MT stability in epidermal cells (Figure 8 and Figure 9). Floating petals on oryzalin-containing solutions for 20 h showed that lipotubuloid movement and structure was not disrupted (Supplementary Video S6). MBD-GFP expression demonstrated that depolymerization of cortical MTs was extensive by 1 h (Figure 8b) and complete within 5 h (Figure 8c). Several hours after oryzalin wash-out began, short cortical MTs were present (Figure 8d) but we failed to observe the recovery of a full network of cortical MTs. While MT labeling in lipotubuloids was absent or rare in controls and during oryzalin treatments (Figure 8a–c), short MTs were prominent within the lipotubuloids during the wash-out (Figure 8d–f) (Supplementary Video S8).

TEM was used to compare the organization of lipotubuloids during oryzalin treatments and during oryzalin wash-out (Figure 9). A comparison to water-only controls (Figure 9a,e) showed few changes in MT organization within the lipotubuloid following treatment with 20 µM oryzalin for either 4 h (Figure 9b,f) or 20 h (Figure 9d,h). MTs were also present in cells that had been washed of oryzalin for 4 h (Figure 9d,h); although, the short MTs seen with MBD-GFP labeling could not be distinguished from MTs that were normally present within the lipotubuloid.

## 3. Discussion

### 3.1. Lipotubuloid Structure

Our live cell analysis with a range of different GFP fusion proteins confirmed the complex structure of the lipotubuloids present in the epidermal cells of *Ornithogalum umbellatum* bracts, petals and sepals. In addition to LDs, the lipotubuloids exhibit a diverse composition of endoplasmic reticulum, Golgi, peroxisomes and mitochondria that typically surround a central vacuole. These observations extend previous TEM studies that were conducted in the ovary cells of *O. umbellatum* [10]. Our use of petal, sepal and bract epidermal cells was advantageous for biolistic transformations, and these whole mount samples proved suitable for live cell imaging with fluorescence and transmitted light. Time-lapse imaging demonstrated that on the rare occasions when cells with multiple lipotubuloids were observed, the lipotubuloids were never observed to merge, and single lipotubuloids were never observed to split. Time-lapse imaging also demonstrated that the lipotubuloids were structurally stable, and that larger organelles such as mitochondria, Golgi or peroxisomes did not move into or out of the lipotubuloids. The presence of these organelles inside lipotubuloids may indicate that they were trapped there as the lipotubuloids formed. Unfortunately, limitations with biolistic transformation approaches meant that our methods were not suitable for studying the early stages of lipotubuloid formation, but the progression of lipotubuloid development has been followed during cell expansion in ovary tissue with the lipotubuloids expanding in size and developing a central vacuole [22].

Time-lapse imaging demonstrated that petal epidermal lipotubuloids undergo rapid cytoplasmic streaming (Supplementary Videos S1 and S2). When quantified, most lipotubuloids moved at between 0.6 and 1.0 μm·s^−1^ but sometimes up to 2.0 μm·s^−1^. Streaming was rapidly inhibited on microfilament disruption with latrunculin and did not recover when latrunculin was washed out of cells but remained unchanged during longer treatments with the MT depolymerizing drug oryzalin (Figure 4, Supplementary Video S2). Immunoelectron microscopy has suggested the presence of both MTs and actin microfilaments inside lipotubuloids [19], and interactions between the microfilaments and the lipotubuloid MTs have been hypothesized to generate lipotubuloid motility [13,19]. We consider this model unlikely. Instead, lipotubuloid motility results from interactions between the long, cortical actin microfilament bundles and the microfilament basket that surrounds the lipotubuloid. The interactions of these microfilaments would drive rotation and streaming in the same way that a similar basket of microfilaments around chloroplasts drives their motion [23].

### 3.2. Lipotubuloid-like Structures in Other Plants

Lipotubuloid-like structures occur in a diverse range of other plant species. The LD aggregates in leaf and stem epidermal cells of the blood lily *Haemanthus albiflos* are spherical shells (5~10 μm diameter) of small droplets surrounding a central vacuole [24], containing other organelles including ER and mitochondria [25], and also have a MT network [26]. Similar LD aggregates occur in root epidermal cells of the hollyhock *Althaea rosea* [27,28], the root epidermal cells of the mallow *Malva neglecta* [27], the leaves of *Vanilla* orchids [25,28,29] and the ovary epidermis of the lily *Hosta sieboldiana* [28], with all these LD aggregates having associations with MTs [28]. MTs are also associated with LDs in the liverwort *Marchantia paleacea* [30], but while LD aggregates are common in liverworts [31], an ultrastructural survey of liverwort species did not detect further examples of MTs associated with LDs [32]. Moreover, these other examples of MTs associated with LDs are less well organized than the MT sheets present in *Ornithogalum*.

### 3.3. Lipotubuloid Functions and the Functions of Lipotubuloid Microtubules

The roles played by *Ornithogalum* lipotubuloids, if any, remain unclear. Biochemical evidence suggests that the lipotubuloids are sites of lipid biosynthesis [15] and that they could contribute to the formation of the cuticle [16,17,18]. We also note that the inclusion of peroxisomes within the lipotubuloids may be significant because of their roles in the b-oxidation of lipids, with physical contact between LDs and peroxisomes often extensive within plant cells [33]. While our research does not address the question of lipotubuloid function, we suggest that the ability to transform *O. umbellatum* epidermal cells and conduct live cell imaging may be critical in clarifying these functions and note that our biolistic transformation approach might be compatible with RNAi-based gene silencing [34,35]. The roles played by lipotubuloid MT would, however, appear to be clearer. Our observations that MT can flatten lipotubuloids, that lipotubuloids are often elongated and that MTs are present within lipotubuloid clefts all suggest that the MTs are exerting a force on the lipotubuloids. Further, the observations suggest that a function of the MT is to prevent the energetically favorable formation of larger LDs by coalescence, thereby retaining maximal LD surface area and facilitating transport of material into and out of them.

### 3.4. Microtubule Organization in Lipotubuloids

The cytoplasmic cortex of *Ornithogalum* sepals and petals contain an extensive MT network which was predominantly organized into transverse arrays, with similar patterns shown by both transient expression of GFP-MBD (Figure 6a and Figure 8a) and immunofluorescence (Figure 6c). These MTs responded rapidly to the presence of oryzalin, undergoing depolymerization. The cortical MT arrays of *Ornithogalum* are, therefore, typical of MTs found in other epidermal cells [36]. Similar cortical MT arrays were also reported in *O. umbellatum* by immunofluorescence [37], although in the absence of confocal imaging in that study, the MTs were difficult to view.

The lipotubuloid MTs show marked differences to the cortical MTs. Neither our immunofluorescence imaging nor our transient expression of GFP-MBD demonstrated extensive MT arrays in lipotubuloids despite TEM showing their presence: previously published immunofluorescence images that were claimed to show lipotubuloid MTs lack visible MTs [37]. Moreover, both immunofluorescence and the expression of GFP-MBD demonstrated that MT depolymerization with oryzalin effectively removed the cortical MT cytoskeleton, but TEM showed that oryzalin failed to disrupt lipotubuloid MTs. Previous studies have also demonstrated the lipotubuloid MTs are more stable during fixation than cortical MTs [37] and that the lipotubuloid MTs were resistant to 6 h-long treatments with 25 µM propyzamide, another MT depolymerizing drug [38]. Furthermore, while we observed MTs with appropriate diameters and rotational symmetry, previous research has suggested variability in MT diameters and the number of protofilaments present [11,39]. Thus, several questions need to be answered about lipotubuloid MTs. First, why are the lipotubuloid MTs stable in the presence of the MT depolymerizing drug oryzalin? And second, why do both immunofluorescence microscopy and GFP fusion proteins fail to report the lipotubuloid MTs? One possible answer to both these questions relates to the stabilization of the lipotubuloid MTs.

The mode of action of oryzalin, and of related MT depolymerization herbicides including propyzamide, can explain how lipotubuloid MTs resist depolymerization. Because oryzalin binds to α-tubulin in an unpolymerized tubulin dimer, preventing its polymerization into a MT [40], oryzalin acts as a MT depolymerization agent only if the MTs are dynamic and turning over. If MTs are stable, oryzalin will not depolymerize MTs. Thus, our experiments showing that oryzalin disrupts cortical MTs but not lipotubuloid MTs might imply that the lipotubuloid MTs are stabilized relative to the cortical arrays. This increased stability of lipotubuloid MTs has been previously recognized during fixation for TEM [35]. This MT stabilization might occur in different ways. Mutations in α-tubulin that prevent or reduce oryzalin binding can generate MTs that are apparently hyperstable to oryzalin. These mutations are often associated with the evolution of resistance to dinitroaniline herbicides in weeds [41,42]. However, these resistance-generating mutations would not necessarily alter the rates of MT turnover on their own, although certain mutations that confer oryzalin resistance do also change MT polymerization dynamics [43]. Instead, these mutations make the tubulin/MTs unresponsive to oryzalin, and normal MT dynamics would still occur. For this mechanism to explain lipotubuloid MT stability in *Ornithogalum*, distinct lipotubuloid tubulin isoforms would be required. Alternatively, MTs might be stabilized through post-translational modifications to the tubulin dimers, with this having been implicated in the stability of neuronal MTs [44]. While both these possible mechanisms would seem unlikely, they cannot be ruled out in the absence of biochemical tests.

Another explanation for the increased stability of lipotubuloid MTs would be the presence of MT stabilizing agents. Based on the staining associated with the surface of lipotubuloid in TEM images, an observation consistent with our own TEM images (Figure 2a), Kwiatkowska and colleagues suggested that polysaccharides covering the MT surfaces might account for their increased stability [37]. Instead, we suggest that MT-associated proteins (MAPs) might bind along the MTs and promote their formation into the extensive bundles and sheets present in lipotubuloids. Extensive binding of the lipotubuloid MTs by MAPs might also explain why these structures were difficult to label with GFP-MBD. This construct, based on the MT-binding domain of mouse MAP4 [20], binds along the sides of MTs. Were these MAP4-binding sites blocked with endogenous lipotubuloid MAPs, then the MAP4-GFP construct would not bind and the MTs would not be visible. Similarly, these endogenous MAPs might occlude antibody binding sites preventing immunolabelling. During oryzalin wash-out, however, MTs repolymerizing within the lipotubuloid would be able to incorporate GFP-MBD into the MT bundles. This would explain why MT labeling within lipotubuloids was only possible after oryzalin recovery. Unfortunately, our attempts to isolate lipotubuloids and identify any MAPs that are present have been unsuccessful because of the high mucilage content within the petals and sepals.

## 4. Materials and Methods

### 4.1. Plant Material

Bulbs of *Ornithogalum umbellatum* L. (Tesselaar, Silvan, VIC, Australia or Yates, Auckland, New Zealand) were grown in pots in greenhouses and in outdoor beds to stagger flowering.

### 4.2. Transient Expression of Fusion Proteins in Epidermal Cells

Plasmid DNA (0.2 to 0.5 μg) coding GFP fusion proteins targeted to different cellular locations (Table 1) was prepared and coated onto gold particles (1.0 or 1.6 μm diameter; Bio-Rad, Regents Park, NSW, Australia) as described previously [45,46,47]. Bombardments used either a PDS-1000 gene gun (Bio-Rad) using 1100 psi rupture discs or a particle inflow gun (Kiwi Scientific, Levin, New Zealand) that used a 30-millisecond pulse of gas at 60 psi. Unopened flowers were removed from the stem, and the lower sides of petals and sepals were bombarded. Transformed flowers were stored in the dark between moistened tissues and viewed after 24 h. Because transformants were incubated overnight, observations were conducted solely on mature flowers.

### 4.3. DIC Video Microscopy

Bracts mounted in water were imaged with a Axioplan microscope (Carl Zeiss, Macquarie Park, NSW, Australia equipped with DIC optics and 40× NA1.4 and 100× NA1.4 lenses. Images were captured with a Sony TRV-900 camera (North Sydney, NSW, Australia).

### 4.4. Confocal Microscopy of Living Material

Living plant material was mounted in distilled water and imaged on a confocal microscope (Leica SP2 or SP5, Wetzlar, Germany) with water or glycerol immersion lenses. GFP and YFP were excited with 488 nm laser light and fluorescence collected from 500 to 550 nm, while RFP was excited at 561 nm with fluorescence collected from 570 to 620 nm. Transmitted light and confocal reflected light images were also collected. LDs were stained with Nile Red (Sigma, St Louis, MO, USA; 15 min, 0.5 μg·mL^−1^) which was excited at 488 nm and imaged from 500 to 600 nm.

MTs were disrupted with oryzalin (Lilley, Greenfield, IN, USA; 20 μM), while actin microfilaments were disrupted with latrunculin B (MP BioMedicals, Sydney, NSW, Australia; 1 μM). All drug and wash solutions were adjusted to 0.5% dimethyl sulfoxide (DMSO). Drug treatments were performed on petals labeled with Nile Red with confocal time-lapse images recorded under set conditions with a 10× lens on randomly selected areas to determine rates of lipotubuloid movement which were quantified in ImageJ.

### 4.5. Immunofluorescence Microscopy

Immunofluorescence protocols were adapted from previously published methods [51]. Petals were fixed in PME solution (50 mM Pipes pH 7.0 K^+^, 2 mM MgSO_4_, 2 mM EGTA) containing 0.1% Triton X-100, 3.7% formaldehyde, 1% glutaraldehyde and 1% DMSO for 1 h and then washed several times in PME containing 0.1% Triton X-100. After extraction in PME containing 1% Triton X-100 (1 h), flowers were washed in phosphate-buffered saline (PBS; 131 mM NaCl, 5.1 mM Na_2_HPO_4_, 1.56 mM KH_2_PO_4_, pH 7.2), extracted in ice-cold methanol (20 min) and washed in PBS. Glutaraldehyde-induced autofluorescence was limited by reduction of free aldehydes with NaBH_4_ (5 mg·ml^−1^; 20 min), and the material was washed in PBS. Cell walls were permeabilized by freeze-fracture [52], and the fragments were blocked in incubation buffer (PBS containing 1% BSA and 0.1% Tween 20). Petals were incubated in mouse monoclonal anti-α-tubulin (1/1 000 in incubation buffer; clone B512; Sigma) overnight at 4 °C, washed several times in PBS and incubated in fluorescein isothiocyanate (FITC)-conjugated sheep anti-mouse IgG (1/50; Silenus-Amrad, Boronia, VIC, Australia). Material was washed in PBS, mounted in AF1 antifade agent (Citifluor, London, UK) and viewed by confocal microscopy with a 63× NA1.25 glycerol immersion lens, with FITC excited at 488 nm and with fluorescence collected from 500 to 550 nm.

### 4.6. Transmission Electron Microscopy

Petals and sepals were cut into thin strips and fixed in PME buffer containing 3% formaldehyde and 1% glutaraldehyde (4 h) during which the samples were evacuated and brought back to air pressure three times. Three PME buffer washes were followed by post fixation (4 h) in buffered 1% osmium tetroxide. Dehydration was in acetone with incremental 20% steps. After a two-step infiltration overnight on a rotator, the samples were embedded in Spurr’s resin at 70 °C. Then, 100 nm thin sections were cut on an LKB Ultrotome, stained with uranyl acetate and lead citrate [53] and examined with an FEI (Charlottesville, VA, USA) Morgagni 268D transmission electron microscope (Hillsboro, OR, USA). MT diameters were measured directly from electron micrographs in ImageJ (version 1.53f51, National Institute of Health, Bethesda, MD, USA).

## 5. Conclusions

The lipotubuloid is an unusual aggregate of LDs enmeshed in MTs that also contains a range of other organelles. While the function of lipotubuloids remains unknown, it is the unusual biochemical properties of lipotubuloid MTs that are the most interesting feature of this structure. While these MTs are readily observed by TEM, they proved to be difficult to image by live cell imaging and immunofluorescence. Moreover, their resistance to the MT depolymerizing herbicide oryzalin suggests that these MTs are hyperstable. Biochemical and gene silencing investigations of the MT-rich *Ornithogalum* lipotubuloids, and the similar structures present in other plant species, might allow identification of novel MAPs and microtubule functions.

## Figures and Tables

**Table 1:** Fluorescent fusion proteins transiently expressed in *Ornithogalum umbellatum* flowers.

Target Organelle	Name	Description	Source	Ref.
cytoplasm	YFP	Free YFP	Madeleine Rashbrooke (ANU)	
mitochondria	AOX-GFP	N-terminal fusion to alternative oxidase	Oliver Berkowitz (ANU)	[48]
endoplasmic reticulum	GFP-HDEL	N-terminal fusion of *Arabidopsis* chitinase signal sequence and C-terminal ER retention motif-HDEL	Jan Marc (Sydney University)	[49]
Golgi apparatus	STtmd-GFP	N-terminal fusion of 52 amino acids of rat sialyltransferase	Daigo Takemoto (ANU)	[21]
microtubules	GFP-MBD	C-terminal fusion to MT-binding domain of mouse MAP4	Jan Marc (Sydney University)	[20]
microfilaments	GFP-hTalin	N-terminal fusion to actin-binding domain of human talin	Daigo Takemoto (ANU)	[21]
tonoplast	ShMTP1-GFP	N-terminal fusion of manganese transporter from the legume *Stylosanthes hamata*	Manny Delhaize (CSIRO Plant Industry)	[50]
peroxisomes	GFP-SKL	C-terminal fusion of tripeptide -SKL, a peroxisomal transport sequence	Robert Mullen (University of Guelph)	[45]
plastids	rbcs-RFP	C-terminal fusion to small subunit of Arabidopsis rubisco	Oliver Berkowitz (ANU)	[48]

## Data Availability

The original contributions presented in this study are included in the article/Supplementary Material. Further inquiries can be directed to the corresponding author.

## Supplementary Materials

The following supporting information can be downloaded at https://www.mdpi.com/article/10.3390/plants14233677/s1: Figure S1: Serial sections through an *O. umbellatum* lipotubuloid showed that microtubule bundles continued between sections; Video S1: DIC video microscopy demonstrated cytoplasmic streaming as well as lipotubuloid rotations. This movie was previously published [12] and appears with permission; Video S2: Lipotubuloid structure and motility were unaffected by floating petals on water and for 20 h on a solution containing 20 µM oryzalin; Video S3: Free YFP demonstrated cytoplasm inside the lipotubuloid; Video S4: The lipotubuloid contains a stable network of endoplasmic reticulum; Video S5: Mitochondrial-targeted GFP demonstrates that the lipotubuloid is a stable structure; Video S6: Peroxisomal-targeted GFP demonstrates that the lipotubuloid is a stable structure; Video S7: Despite providing extensive labeling of cortical microtubules, there was little GFP-MBD label present inside lipotubuloids; Video S8: Extensive labeling of short microtubules with GFP-MBD occurs inside the lipotubuloid during recovery from oryzalin.

## Abbreviations

The following abbreviations are used in this manuscript:

DIC	differential interference contrast
DMSO	dimethyl sulfoxide
GFP	green fluorescent protein
LD	lipid droplet
MAP	microtubule-associated protein
MT	microtubule
RFP	red fluorescent protein
TEM	transmission electron microscopy
YFP	yellow fluorescent protein
