# Supplementary figures and images for: The Lipotubuloids of Ornithogalum umbellatum L. Contain Hyperstable Microtubules
Source: Plants (Basel). 2025 Dec 3;14(23):3677. doi: 10.3390/plants14233677 (PMC12693804; doi:10.3390/plants14233677)

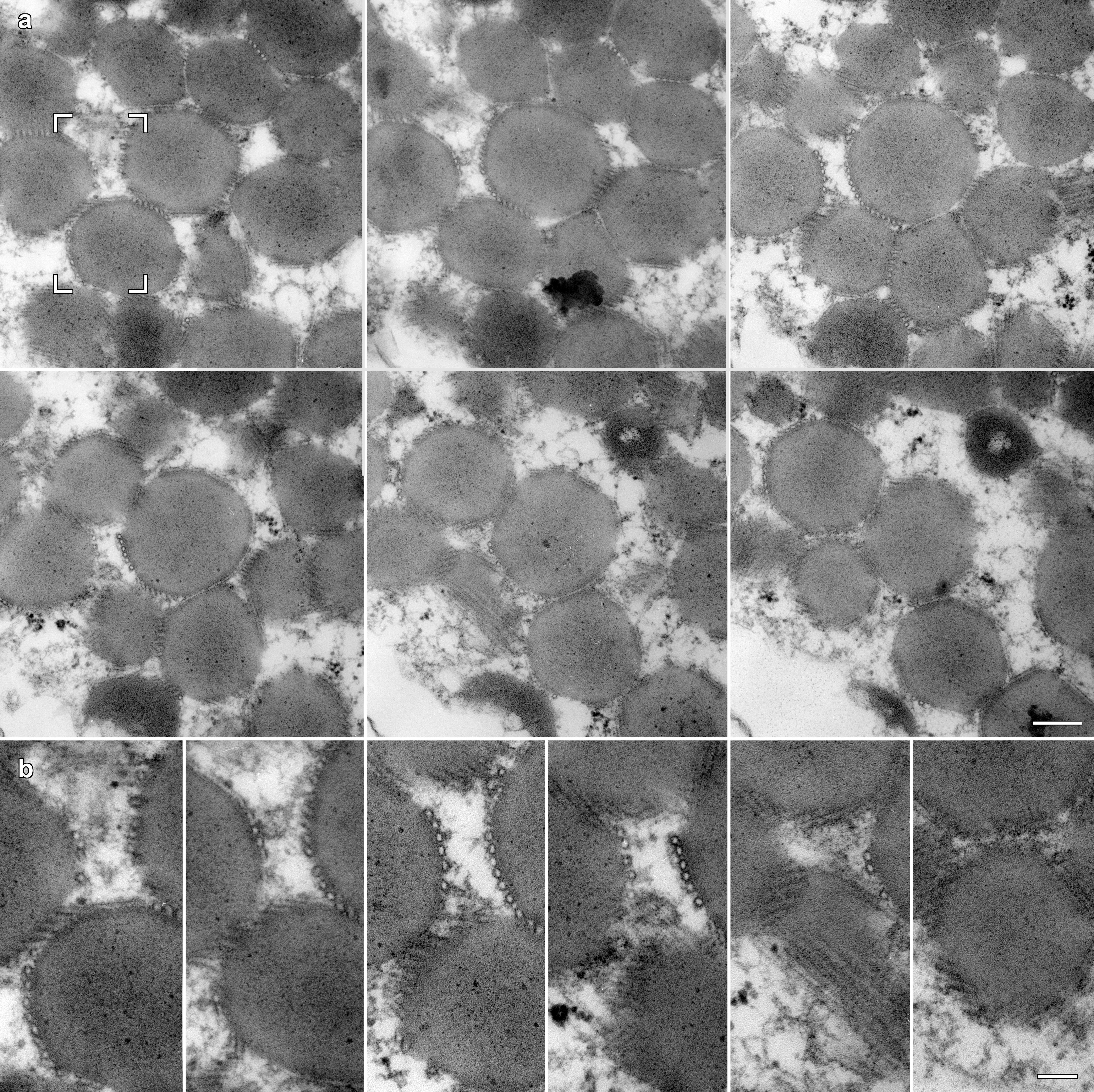

Supplement: Supplementary file 1 [file plants-14-03677-s001.zip › Figure S1.jpg]
